# COVID-19 vaccines: breaking record times to first-in-human trials

**DOI:** 10.1038/s41541-020-0188-3

**Published:** 2020-04-30

**Authors:** Young Chan Kim, Barbara Dema, Arturo Reyes-Sandoval

**Affiliations:** The Jenner Institute, Nuffield Department of Medicine, University of Oxford, Oxford, UK

**Keywords:** Viral infection, RNA vaccines

## Abstract

The twenty-first century has come with a new era in vaccinology, in which recombinant genetic technology has contributed to setting an unprecedented fast pace in vaccine development, clearly demonstrated during the recent COVID-19 pandemic.

COVID-19 is caused by the Severe Acute Respiratory Syndrome coronavirus 2 (SARS-CoV-2). As of 15th April 2020, the World Health Organization (WHO) has reported over 1.7 million cases of COVID-19 and 100,000 deaths worldwide^1^. The virus can be transmitted by patients with or with no symptomatology, thus making the control of this disease outbreak a challenging task due to the lack of a specific treatment or vaccine^2^. Without an efficacious licensed vaccine, control of the pandemic relies on self-isolation to prevent close contact with other people and basic measures such as hand washing. Quarantine is efficacious but causes major disruption to the economy of people and countries^3^. Therefore, development of a safe and effective vaccine against COVID-19 is an urgent public health priority.

Over the last century, control of epidemics has been achieved successfully thanks to vaccines developed using various technologies, predominantly by classic pathogen inactivation or attenuation. This has worked efficiently for Cholera, Typhoid, Polio, Measles, Plague or Tetanus. Conjugate-vaccines and subunit vaccines have also provided effective triumphs in vaccinology for pneumonia, sepsis and meningitis^4^. The pace of these vaccine developments is comparatively slow to that imprinted by 21st-century vaccines that use recombinant genetic technology. During the recent pandemic of COVID-19, six vaccine candidates encoding or presenting SARS-CoV-2 antigens have entered phase I clinical trials to assess their safety and immunogenicity, including those based on mRNA (NCT04283461), adenoviral vector 5 (NCT04313127); chimpanzee adenoviral vector ChAdOx1 (NCT04324606), DNA (NCT04336410), a lentiviral vector (NCT04276896) and artificial antigen-presenting cells or aAPC (NCT04299724). Despite the fact that most of these COVID-19 vaccine candidates are being evaluated in phase I trials, some are experimental (DNA/RNA vaccines) and may have a longer journey ahead to achieve licensure. Available information indicates that various candidates express the COVID-19 spike (S) glycoprotein to neutralise the virus and prevent attachment to the human angiotensin converting enzyme II (ACE2) receptor, known to be the co-receptor for viral entry of SARS-CoV-2^5^.

The mRNA1273-COVID-19 vaccine has set a record time by reaching trials (NCT04283461) in only 69 days after the identification of the SARS-CoV-2 as the causative agent of the current outbreak^6^. This is a nanoparticle encapsulated (LNP) mRNA vaccine that encodes a full length, prefusion stabilised spike (S) glycoprotein, which progressed directly to clinical assessment without pre-clinical studies due to its potentially safe nature, thus accounting for its speed in reaching phase I trials. A recombinant novel COVID-19 vaccine based on an adenovirus vector 5 (Ad5-nCoV) encoding the full-length S protein has progressed fastest and has now entered phase II trials from 12th April 2020. The INO-4800, DNA plasmid-based vaccine encodes the S protein and is delivered by two intradermal injections followed by electroporation of the DNA vaccine in healthy volunteers. The COVID-19 specific aAPC vaccine has been prepared by transfection of aAPCs with a genetically-modified lentivirus encoding the SARS-CoV-2 structural and protease protein domains to aAPCs, which are delivered by three subcutaneous injections to healthy and COVID-19 positive volunteers between age of 6 months to 80 years. The lentiviral-based COVID-19 (LV-DC) vaccine and antigen-specific cytotoxic T cell (CTL) vaccine encoding COVID-19 antigens were given via subcutaneous injection and intravenous (IV) infusion respectively to the volunteers including the laboratory (RT-PCR) confirmed COVID-19 infections as part of Phase I/II trial. In addition, a COVID-19 vaccine based on Chimpanzee Adenovirus Vector (ChAdOx1) developed by University of Oxford has entered phase I/II clinical trial in April 2020 to test its safety, tolerability and reactogenicity profile, as well as its immunogenicity in 510 volunteers. This vaccine also aims to be assessed for efficacy to prevent infection measured by PCR as well as symptomatic infection (NCT04324606). Chimpanzee adenoviral vectors are replication-deficient vaccines that carry one or a few encoded antigens and efficiently stimulate both arms of the adaptive immune responses: humoral and cytotoxic T-cells (CTLs). They have been very well-studied as a vaccine platform in over 10 different pathogens with safe profile in thousands of volunteers from 1 week of age to 90 year-old volunteers^7^.

In comparison, other Coronaviruses such as MERS-CoV^8^ and SARS-CoV^9^ have reached clinical trials within ~22 months and ~25 months, respectively after their outbreaks (NCT02670187, NCT00099463). Both first clinical trials were based on DNA vaccines encoding the spike (S) glycoprotein and even though the results of the SARS-CoV vaccine have not been published yet, MERS-CoV DNA vaccine preliminary results showed good tolerability and immunogenicity in humans, with immune responses similar to the ones elicited after natural infection, which supports further development.

This pace of development is striking when compared to new emerging diseases causing major epidemics declared by the WHO such as the arboviral diseases Dengue^10^, Chikungunya^11,12^ and Zika^13–15^, which reached trials in 52, ~19 and ~9 years after declaration of major outbreaks, respectively ((13), (17), NCT02840487). Dengue Virus has been in circulation for more than a century and a tetravalent live attenuated vaccine produced by Sanofi Pasteur has only been authorized by the European Medicine Agency in 2018. However, the very first clinical trial was done during the World War II by Albert Sabin, who used a Dengue virus originally attenuated in mice^16^. The first chikungunya vaccine tested in humans in the late 60’s used a formalin-inactivated virus^17^, which was subsequently abandoned for new vaccine platforms such as a virus-like particle particle (VLP) platform (NCT01489358), which has showed high titters of neutralizing antibodies in recipients after a second dose^18^. The first Zika DNA vaccine reached trials in August 2016, 9 years after ZIKV outbreak in part of the Federated States of Micronesia, 3 years after the major epidemic in French Polynesia in October 2013 but just 6 months after WHO declaration as the Public Health Emergency of International Concern (PHEIC) on February 2016, highlighting the advances in the modern vaccine development in urgent need. A Zika DNA vaccine delivered in a split-dose needle freeway, was able to induce six times higher immune responses compared to a single-dose delivery via needle and syringe and therefore moved into an international placebo-controlled phase 2 efficacy trial^19^.

Other emerging diseases that have caused major epidemics are Haemorrhagic fever viral diseases such as Ebola^20^, Crimean-Congo fever^21^ and Lassa fever^22^. These have taken more than three decades to get to the first-in-human assessment (NCT00072605, NCT03020771, NCT03805984) (Fig. 1) but unfortunately not all of them have described safety and tolerability results yet. However, the rVSV-Ebola vaccine candidate based on a live, attenuated recombinant vesicular stomatitis virus vector produced by Merck has progressed beyond I/II/III clinical trials^23,24^ receiving approval by the US FDA^25^ in December 2019; whereas a viral-vectored Ebola vaccine candidate consisting of Ad26/MVA has now completed phase III trial (NCT02543567). Initial vaccine clinical trials may not always lead to a successful license but can pave the way to the success of future vaccines in acquiring the license.

**Fig. 1:**
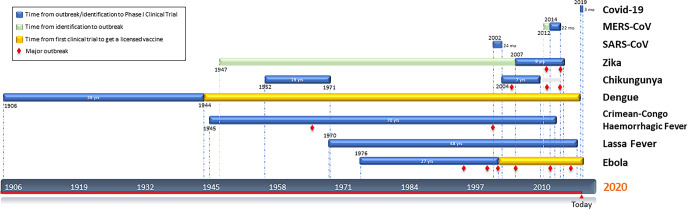
New emerging diseases vaccine development timeline. Time since the first identification (green) of the virus in a patient or first major outbreak (blue) (and in some cases newly identification) to first Phase I Clinical Trial described. Orange bar represents the time it took to get a licensed Dengue/Ebola vaccine. Red diamonds indicate outbreaks.

The great majority of licensed vaccines are based in inactivation/attenuation pathogens which lengthen the development, cost and production of the vaccine. Recombinant viral vectored, DNA/RNA and protein technologies are setting the fastest records in vaccine development but just a selected few have been licensed so far for veterinary use only, since, for humans some vaccines have not met some regulatory requirements for approval and commercialization yet but international emergencies like the current COVID-19 could provide a final push towards obtaining licensure. This highlights the potential of vaccinology to make fast progress when appropriate international support exists, proving that when there is a will, there is a way.
